# Congenital fistulisation of Meckel’s diverticulum in omphalocele sac: case report

**DOI:** 10.11604/pamj.2019.32.20.15010

**Published:** 2019-01-15

**Authors:** Mohammed Tazi Charki, Hicham Abdellaoui, Saad Andaloussi, Mohammed Amine Oukhouya, Abdelhalim Mahmoudi, Aziz El madi, Khalid Khattala, Youssef Bouabdallah

**Affiliations:** 1 Department of Pediatric Surgery, University Hospital of Hassan II, University of Sidi Mohamed Ben Abdellah, Fez, Morocco

**Keywords:** Meckel’s diverticulum, exomphalos, fistulisation

## Abstract

Fistulisation of Meckel's diverticulum in the top of an omphalocele sac is very rare. To our Knowledge, three cases were reported in the literature. We presente in this report a new case of this uncommon presentation.

## Introduction

Minor degrees of omphalocele have been known to be associated with vitellointestinal duct (VID) anomalies. The most frequent association is Meckel's diverticulum (MD) [1]. But the fistulisation of the diverticulum in the top of the omphalocele sac is very rare. To our Knowledge, three cases were reported in the literature [2-4]. Herein, we report a new case of this uncommon presentation.

### Patient and observation

**Figure 1:**
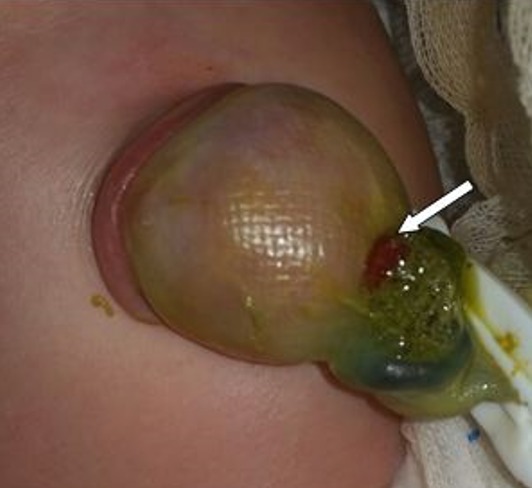
Omphalocele with fistula draining a greenish meconium (white arrow)

**Figure 2:**
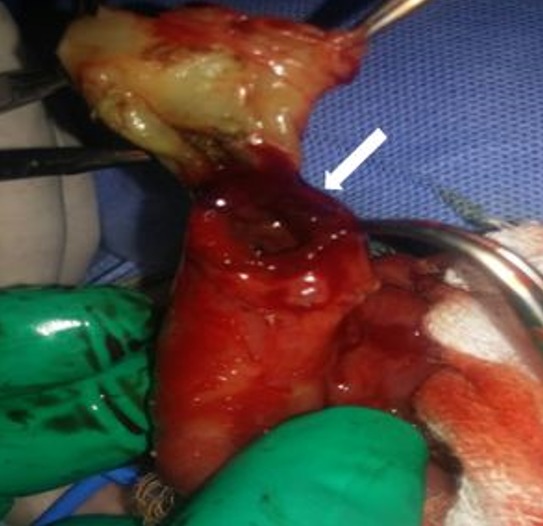
Peroperative picture showing fistulisation of a Meckel’s diverticulum adhered to the omphalocele sac. (white arrow)

**Figure 3:**
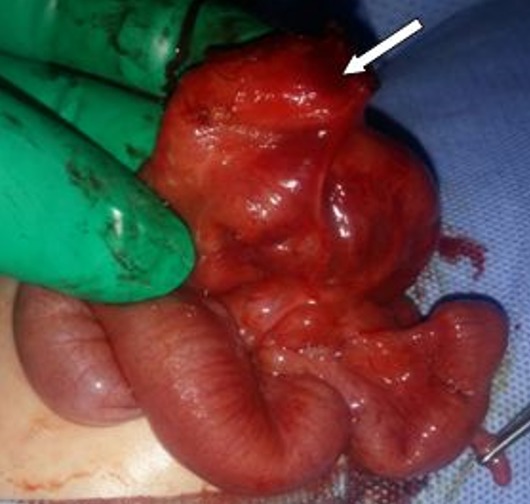
Peroperative picture showing Meckel’s diverticulum located at the 4th intestinal loop before Bauhin valve (white arrow)

A one-day old, full term male, was referred with a swelling at umbilical region. Prenatal ultrasonography was not performed. Birth weight was 3500g. In physical examination he had normal morphological features. There was an omphalocele making 6cm with a collar of 4cm, covered with a thin, semi-transparent membrane with fistula draining meconium (Figure 1). The umbilical cord above the omphalocele sac measured approximately 2cm. The small bowel loops were visible through the thin membrane. Rest of the neonatal examination was unremarkable. Echocardiography was normal. After optimization of the general condition, surgery was performed. After excision of the membrane, exploration found a fistulisation of a Meckel's diverticulum (MD) adhered to the omphalocele sac. The diverticulum size was 2.5cm, located at the 4^th^ intestinal loop before Bauhin Valve (Figure 2, Figure 3). There were no meconium in peritoneal cavity. A resection of the loop including the MD was performed with end-to-end anastomosis. The post operative was simple; breastfeeding was started on the 4^th^ day after surgery and meconium was emitted by the anus. The newborn was discharged without complication. Histopathology of the excised specimen revealed wall of small intestine without ectopic mucosa.

## Discussion

Omphalocele is a midline abdominal wall defect in which a thin membrane surrounds the protruding organs that can include small intestine, liver, bladder, spleen, stomach, uterus and ovaries [5]. It occurs in 1 in 4,000 to 6,000 live births [6] and can be associated to others abnormalities in 50 to 75 % of cases, including heart defects, cleft lip or palate, intestinal, vesical, genital, or diaphragmatic malformations; and chromosomal anomaly [7, 8]. Small omphalocele size is associated with fewer cardiac anomalies but with an increased prevalence of gastrointestinal anomalies such as ileal or colonic atresia and VID abnormalities [9]. In fact, minor degrees of omphalocele have been known to be associated with VID abnormalities, the most frequent is MD [1]. In review of 49 omphalocele, MD was found in 8 cases (16%), the incidence was higher in small omphalocele (28%) than large one (4%) [10]. Prenatal discovery of an omphalocele associated with an inner umbilical cord MD was described in only one case [8]. In our case, we described a fistulisation of MD in the top of omphalocele sac. To our Knowledge, only 3 similar cases were reported in the literature [2-4]. In all cases, omphalocele was minor and resection of the loop including MD with end-to-end anastomosis was performed. Other cases of congenital intestinal fistulisation in minor exomphalos were reported. In the series of Ng J *et al.* about 5 cases on 2006, there were two cases of a patent VID with a fistula, one case of ileal prolapse and in two cases there was a fistulous communication of the ileum directly with the exomphalos sac [1]. In these two cases, the authors suggest a prolapsed of ileum through a patent VID which subsequently underwent spontaneous regression.

## Conclusion

In summary, if fistula is noted in an omphalocele sac, a co-existing patent VID should be considered. The treatment involves a resection of small bowel including the VID anomalies with end-to-end anastomosis.

## Competing interests

The authors declare no competing interests.

## References

[1] Ng J, Antao B, Mackinnon E (2006). Congenital intestinal fistula with exomphalos minor. Pediatr Surg Int.

[2] Jin H, Han J-W, Oh C, Kim H-Y, Jung S-E (2017). Perforated Meckel's diverticulum in omphalocele. J Pediatr Surg Case Rep.

[3] Hale PC, Agrawal M (1993). Congenital fistulation of a Meckel's diverticulum to the surface of an exomphalos sac. Br J Clin Pract.

[4] Mavridis G, Livaditi E, Vassiliadou E, Christopoulos-Geroulanos G (2008). Intrauterine fistulation of perforated Meckel's diverticulum to the surface of the sac of an intact exomphalos minor. Minerva Pediatr.

[5] Corey KM, Hornik CP, Laughon MM, McHutchison K, Clark RH, Smith PB (2014). Frequency of anomalies and hospital outcomes in infants with gastroschisis and omphalocele. Early Hum Dev.

[6] Laughon M, Meyer R, Bose C, Wall A, Otero E, Heerens A (2003). Rising birth prevalence of gastroschisis. J Perinatol Off J Calif Perinat Assoc.

[7] Tourne G, Chauleur C, Varlet MN, Tardieu D, Varlet F, Seffert P (2007). Prenatal discovery of an omphalocele associated with an inner umbilical cord Meckel's diverticulum. J Matern-Fetal Neonatal Med Off J Eur Assoc Perinat Med Fed Asia Ocean Perinat Soc Int Soc Perinat Obstet.

[8] Stoll C, Alembik Y, Dott B, Roth M-P (2008). Omphalocele and gastroschisis and associated malformations. Am J Med Genet A.

[9] Kumar HR, Jester AL, Ladd AP (2008). Impact of omphalocele size on associated conditions. J Pediatr Surg.

[10] Nicol JW, MacKinlay GA (1994). Meckel's diverticulum in exomphalos minor. J R Coll Surg Edinb.

